# Live and Non-Live Pregnancy Outcomes among Women with Depression and Anxiety: A Population-Based Study

**DOI:** 10.1371/journal.pone.0043462

**Published:** 2012-08-24

**Authors:** Lu Ban, Laila J. Tata, Joe West, Linda Fiaschi, Jack E. Gibson

**Affiliations:** 1 Division of Epidemiology and Public Health, Nottingham City Hospital, University of Nottingham, Nottingham, United Kingdom; 2 Nottingham Digestive Diseases Centre National Institute for Health Research Biomedical Research Unit, Queen's Medical Centre, Nottingham University Hospitals National Health Service Trust, Nottingham, United Kingdom; Vanderbilt University, United States of America

## Abstract

**Background:**

Women taking antidepressant or anti-anxiety medications during early pregnancy have high risks of non-live pregnancy outcomes, although the contribution of the underlying illnesses to these risks remains unclear. We examined the impacts of antenatal depression and anxiety and of commonly prescribed treatments on the risks of non-live pregnancy outcomes.

**Methods:**

We identified all pregnancies and their outcome (live birth, perinatal death, miscarriage or termination) among women aged 15–45 years between 1990 and 2009 from a large primary care database in the United Kingdom. Women were grouped according to whether they had no history of depression and anxiety, a diagnosis of such illness prior to pregnancy, illness during pregnancy and illness during pregnancy with use of medication (stratified by medication type). Multinomial logistic regression models were used to compare risks of non-live outcomes among these groups, adjusting for major socio-demographic and lifestyle characteristics.

**Results:**

Among 512,574 pregnancies in 331,414 women, those with antenatal drug exposure showed the greatest increased risks for all non-live pregnancy outcomes, relative to those with no history of depression or anxiety, although women with prior (but not currently medicated) illness also showed modest increased risks. Compared with un-medicated antenatal morbidity, there was weak evidence of an excess risk in women taking tricyclic antidepressants, and stronger evidence for other medications.

**Conclusions:**

Women with depression or anxiety have higher risks of miscarriage, perinatal death and decisions to terminate a pregnancy if prescribed psychotropic medication during early pregnancy than if not. Although underlying disease severity could also play a role, avoiding or reducing use of these drugs during early pregnancy may be advisable.

## Introduction

The use of selective serotonin reuptake inhibitors (SSRIs) and other antidepressants, primarily for depression and anxiety, has increased dramatically in the last two decades [1] and 5% of pregnant women may use these medications during the first trimester of pregnancy. [2] Prior research suggests that taking antidepressants during early pregnancy has adverse effects on pregnancy outcomes and neonatal health, [3] including increased risks of miscarriage [4]–[9] and perinatal death. [5], [10] It has also been suggested that women taking antidepressants during pregnancy are more likely to have a termination, [5], [9], [11] however the majority of these studies have been small-scale and few have considered the contribution of the underlying mental health conditions which necessitate treatment to the observed effects. [12] It has been well documented that women with antenatal depression or anxiety have increased risks of a range of adverse pregnancy complications, [13] such as preeclampsia, [14] preterm delivery, [15] and prolonged labour. [16] Previous research has also highlighted associations between mental illness and maternal smoking [17] and abnormal endocrine and immune regulation, [18] which may directly impair the development of the foetus and lead to foetal growth retardation and low birth weight. [19] The observed increased risks of non-live pregnancy outcomes could therefore be at least partly explained by the mental health conditions themselves, as well as by associated lifestyle characteristics of these women. Indeed, a recent large population-based study [20] in Sweden found no association between the use of antidepressants during early pregnancy and the risk of perinatal death after controlling for maternal socio-demographic characteristics.

In contrast to antidepressants, benzodiazepines and other anti-anxiety drugs (also commonly prescribed to women of childbearing age) have attracted less attention in the literature. Prior research has demonstrated that the use of benzodiazepines during pregnancy is associated with increased risks of adverse neonatal outcomes such as preterm delivery and low birth weight. [21] In addition, two studies in the 1990s showed an association between exposure to benzodiazepines during pregnancy and increased risks of non-live pregnancy outcomes, [22], [23] however these findings were based on small sample populations and the findings have not been widely replicated. In addition, few of the studies of the effects of antidepressant and anti-anxiety drugs have directly compared women with antenatal depression or anxiety who do not use medication with those who do, nor have they sought to examine the effect of drug discontinuation on non-live birth outcomes or to discriminate between different classes of antidepressant and anti-anxiety drugs.

We therefore conducted a large population-based cohort study using data from primary care practices in the United Kingdom (UK) to assess the risks of perinatal death, miscarriage and termination in women with prior or current antenatal depression and anxiety compared with those in women with no history of such illness. We also compared outcomes among women taking psychotropic medications during the first trimester of pregnancy with those among women with depression or anxiety who did not receive medication. We assessed the risks associated with each drug class separately and also investigated whether there was any risk modification if women discontinued these medications at the start of pregnancy.

## Methods

### Data source and ethics statement

We used data from The Health Improvement Network (THIN), a nationally representative database of computerised primary care medical records containing validated recordings of medical diagnoses, events, symptoms and drug prescriptions [24] collected at 446 general practices (primary health care units) throughout the UK. These data are primarily collected and recorded for the purpose of routine management of patient health care in the UK National Health Service (NHS) general practice setting, rather than for research purposes. NHS general practices contributing data to THIN provide consent for the use of these data by researchers. Whilst ethical approval is required for each study using THIN data, direct consent from individual patients is not required under the UK Data Protection Act because all data are anonymised, such that individual patients as well as the names and specific locations of general practices are withheld from researchers. Ethical approval for this study was obtained from the UK Medical Research Ethics Committee (administered and approved by the National Health Service South East Research Ethics Committee; REC reference 04/MRE01/9).

### Study population

From THIN, we identified all clinically recognised singleton pregnancies between 1990 and 2009 and among women aged 15–45 years that ended in live birth, stillbirth, or miscarriage. We also identified all pregnancies that ended in termination and included them in the overall study population. For pregnancies ending in a live birth, we searched the records of both mothers and offspring (linked by delivery details and encrypted residential address) for recordings of infant death within 28 days postpartum, and combined these with stillbirths as a measure of perinatal death. Since the legislation on termination of pregnancy in Northern Ireland is more restrictive than that in other parts of the UK, we excluded women registered at general practices in this province. We also excluded all women with evidence of bipolar disorder, schizophrenia and other psychotic disorders, who comprised less than 0.5% of the original study population.

### Exposure definition

Depression, anxiety and exposure to medication were defined according to the presence or absence of a relevant recording in each woman's primary care electronic health records within the first 90 days following the estimated date of conception (the first trimester of pregnancy). Dates of conception were estimated based on a range of recordings relating to pregnancy (including expected delivery dates, maturity estimates and timing of routine monitoring events), and where no information was available, live births were assumed to take place at 40 weeks and miscarriage and termination at 10 weeks. We extracted records of prescriptions of all antidepressants, hypnotics, and anxiolytics that are primarily indicated for the treatment of depression or anxiety according to British national guidelines. [25] To minimise the risk of detecting reverse-causal effects (where a non-live outcome may be the trigger for depression or anxiety and its treatment), we excluded prescriptions and diagnoses within the last seven days of pregnancies which ended within the first trimester.

We grouped mothers into eight mutually exclusive categories according to their diagnostic and treatment status:

**Group 0.** No history of anxiety or depression (non-exposed group).
**Group 1.** History of diagnosis of anxiety or depression before pregnancy but no diagnostic recordings during the first trimester.
**Group 2.** Diagnostic records of anxiety or depression but no prescriptions of interest during the first trimester.
**Group 3.** Prescriptions for any tricyclic antidepressants (TCAs) (alone - i.e. no other psychotropic medication of interest) during the first trimester.
**Group 4.** Prescriptions for any SSRIs (alone) during the first trimester.
**Group 5.** Prescriptions for any benzodiazepines (alone) during the first trimester.
**Group 6.** Prescriptions for any other single class of drug from the following groups during the first trimester
  1. Other sedative medications: buspirone, meprobamate, zaleplon, zolpidem tartrate, zopliclone, zopliclone, chloral hydrate, triclofos sodium;
  2. Monoamine oxidase inhibitors: phenelzine, isocarboxazid, tranylcypromine and moclobemide;
  3. Other antidepressants: duloxetine, mirtazapine, reboxetine, tryptophan and venlafaxine;
**Group 7.** Prescriptions for two or more classes of psychotropic drug (mentioned above) during the first trimester.

### Co-variables

We identified potential confounders by extracting data on the following characteristics of women: maternal age at the end of pregnancy, the most recent recording of smoking status before delivery, body mass index (BMI, kg/m^2^) before pregnancy and quintiles of Townsend's Index of Deprivation [26] for each woman's postcode of residence. Since women aged 15–17 may have different risks of non-live pregnancy outcomes from older women, [27] we categorised maternal age as follows: 15–17 years, 18–24 years, 25–34 years and 35–45 years. In addition, since women's prior pregnancy history could affect the risk of subsequent pregnancy loss, or of developing mental illness during later pregnancies, [28], [29] we also extracted information on the number of previous known live births (a proxy of parity) for each pregnancy.

### Statistical analyses

Multinomial logistic regression models were used to obtain relative risk ratios (RRRs) for perinatal death, miscarriage and termination relative to live births in each of the seven exposure groups, compared with women without any indication of current or prior depression or anxiety. We included more than one pregnancy for some women and a cluster correction on the women's unique identification codes was applied. To identify potential confounders, chi-squared tests were used to determine whether maternal age, Townsend deprivation index (in quintiles), maternal smoking history or BMI were associated with each exposure, or with any adverse pregnancy outcome among women in the referent group. Co-variables with statistically significant associations at the 5% level with both were included in multivariable models to obtain adjusted RRRs. Missing values for co-variables were fitted as a separate category in the analyses to provide an implicit adjustment for any dissimilarity between women associated with differential recording.

We additionally assessed prior pregnancy history by using chi-squared tests to examine whether the number of previous live births was associated with exposures or with current adverse pregnancy outcomes. We also carried out sensitivity analyses by adding the number of known prior pregnancies into our main multivariable model. Our data were open cohort data that included all prospectively recorded pregnancy outcomes from the point at which women registered with their general practitioner (primary care physicians), which could be at any age during the potentially fertile period. Although all women in the UK must be registered with a general practitioner to receive obstetric care, people do change general practitioners, often because they move home. We could not, therefore, be certain of a complete pregnancy history for all women, particularly for older women. Therefore, we also adjusted for parity in a further multivariable model restricted to women who were registered by the age of 20 in an attempt to minimise misclassification due to unrecorded prior births.

#### Assessing risks of medication use in women with depression or anxiety only

To determine whether the use of psychotropic medication was associated with an excess risk of each adverse pregnancy outcome compared with un-medicated depression or anxiety, we repeated our analyses excluding women without current depression or anxiety (i.e. we excluded the original referent group and group 1), so that RRRs were in reference to group 2 (a recording of depression or anxiety, but no prescriptions during the first trimester).

#### Assessing effect of continued medication use in pregnancy

To investigate whether the risks of adverse pregnancy outcomes in women who continued to receive psychotropic medications after conception were greater than among those who discontinued their use, we carried out a further sensitivity analysis. All women exclusively prescribed any TCAs, SSRIs or benzodiazepines (the three most common medication classes) within 90 days before pregnancy were identified. For each drug class, a multinomial logistic regression model was used to compare the outcomes among women who received a repeat prescription for a drug in the same class during the first trimester of pregnancy with those who did not.

In recognition of the large number of categorisations in each analysis, 99% confidence intervals (CIs) were calculated for each measure of association, and exact (3 dp) p-values are given. All analyses were carried out using Stata SE 11.0 (Stata Corp., TX, USA) for Windows 2007 Enterprise Edition (Microsoft Corporation, Seattle, USA).

## Results

From THIN, we identified 512,574 pregnancies among a cohort of 331,414 mothers. More than half of women were aged 25–34 years and 0.4% of their pregnancies ended in perinatal death (stillbirth or neonatal death), 12.6% in miscarriage and 14.7% in termination (Table 1). Pregnancies ending in terminations were more likely to be in younger women with a history of smoking and from socio-economically deprived groups whilst miscarriage was more common in older women, compared with pregnancies ending in live births. Pregnancies ending in perinatal death were also more likely to occur in women from deprived groups and in those who were overweight or obese, compared with live-birth pregnancies.

**Table 1:** Maternal characteristic for all pregnancy outcomes.

	All pregnancies		Live birth		Perinatal death^a^		Miscarriage		Termination	
Basic characteristics	N = 512,574		n = 370,443		n = 2,096		n = 64,511		n = 75,524	
	n	%	n	%	n	%	n	%	n	%
**Maternal age** at the end of pregnancy, years										
15–17	10,252	2.0	3,708	1.0	22	1.1	1,166	1.8	5,356	7.1
18–24	109,793	21.4	69,495	18.8	390	18.6	11,568	17.9	28,340	37.5
25–34	282,006	55.0	220,642	59.6	1,140	54.4	31,832	49.3	28,392	37.6
35–45	110,523	21.6	76,598	20.7	544	26.0	19,945	30.9	13,436	17.8
**Townsend deprivation index**										
1 (least deprived)	117,018	22.8	88,535	23.9	387	18.5	14,920	23.1	13,176	17.5
2	96,618	18.9	71,566	19.3	342	16.3	12,346	19.1	12,364	16.4
3	100,527	19.6	72,180	19.5	399	19.0	12,743	19.8	15,205	20.1
4	97,608	19.0	68,643	18.5	429	20.5	11,961	18.5	16,575	22.0
5 (most deprived)	74,482	14.5	51,287	13.8	425	20.3	9,030	14.0	13,740	18.2
Missing	26,321	5.1	18,232	4.9	114	5.4	3,511	5.4	4,464	5.9
**Maternal history of smoking** before delivery	208,302	40.6	145,953	39.4	915	43.7	26,616	41.3	34,818	46.1
**Maternal BMI** before pregnancy (kg/m^2^)										
Normal (18.5–24.9)	227,820	44.5	166,999	45.1	787	37.5	28,696	44.5	31,338	41.5
Under-weight (<18.5)	17,485	3.4	12,223	3.3	66	3.2	2,195	3.4	3,001	4.0
Over-weight(25–29.9)	87,909	17.2	65,033	17.6	435	20.8	11,797	18.3	10,644	14.1
Obese (30–39.9)	49,594	9.7	36,561	9.9	284	13.6	7,144	11.1	5,605	7.4
Missing	129,766	25.3	89,627	24.2	524	25.0	14,679	22.8	24,936	33.0

a Stillbirth or neonatal death within the first 28 days postpartum.

BMI = body mass index.

Pregnancies ending in adverse outcomes were more common in all exposure groups compared with the referent group of women with no current or past depression or anxiety (Table 2). The prevalence of miscarriage and perinatal death was highest among women prescribed psychotropic drugs, especially those receiving benzodiazepines, the less common medications (Group 6) and those receiving multiple classes of medication. In women prescribed benzodiazepines only, 0.7% of pregnancies ended in perinatal death and 16.2% in miscarriage. The equivalent proportions for women with un-medicated depression or anxiety were 0.6% and 12.1%, and for those in the referent group were 0.4% and 12.1% respectively (Table 2). In addition, greater proportions of women terminated their pregnancies if they were exposed to psychotropic medication during early pregnancy.

**Table 2:** Breakdown of live and non-live pregnancy outcomes by different antenatal diagnostic and drug exposures.

Mental illness/drug exposures^a^			Live birth		Perinatal death		Miscarriage		Termination	
			N = 370,443		N = 2,096		N = 64,510		N = 75,524	
Referent category^b^	n	(%)	287,814	(73.7)	1,474	(0.4)	47,258	(12.1)	54,119	(13.9)
History of mental illness only	n	(%)	69,297	(69.0)	480	(0.5)	13,814	(14.0)	16,341	(16.5)
Unmedicated mental illness	n	(%)	2,640	(72.4)	20	(0.6)	442	(12.1)	545	(14.9)
TCAs	n	(%)	1,983	(65.7)	18	(0.6)	443	(14.7)	575	(19.1)
SSRIs	n	(%)	6,205	(60.2)	57	(0.6)	1,539	(14.9)	2,511	(24.4)
Benzodiazepines	n	(%)	1,416	(59.4)	16	(0.7)	386	(16.2)	566	(23.7)
Any other single class	n	(%)	645	(54.8)	14	(1.2)	223	(18.9)	296	(25.1)
Multiple classes	n	(%)	1,443	(59.2)	17	(0.7)	406	(16.7)	571	(23.4)

a Exposures were depression or anxiety with or without exposures to different classes of antidepressants or anti-anxiety drugs. All categories were mutually exclusive.

b Reference was no history of or current depression or anxiety.

TCAs = tricyclic antidepressants.

SSRIs = selective serotonin reuptake inhibitors.

Table 3 presents the relative risk ratios for each adverse outcome for each exposure category compared with the referent group. Since the unadjusted and multivariable models produced nearly identical effect estimates, we present adjusted results only. Compared with women from the referent group, women with a history of depression or anxiety and exposure to psychotropic medication during the first trimester of pregnancy had consistently increased risks of all non-live pregnancy outcomes. Effect estimates for exposures to different drugs (especially to SSRIs, benzodiazepines and the less common drug classes, and to multiple classes) were greater than those for un-medicated current illness or for a historical depression or anxiety diagnosis. The greatest effects were found in women prescribed the less common medications (Group 6: adjusted RRRs = 3.7, 2.0 and 2.6, 99% CIs 1.9–7.5, 1.7–2.5 and 2.1–3.1 for the risks of perinatal death, miscarriage and termination, respectively) (Table 3).

**Table 3:** Adjusted relative risk ratios of each adverse pregnancy outcome relative to live birth in each antenatal diagnostic and drug exposure category compared with no current/past depression or anxiety (512,574 pregnancies in 331,414 women).

	Perinatal death		Miscarriage		Termination	
Mental illness/drug exposures^a^	n = 2,096		n = 64,510		n = 75,524	
	RRR^c^ (99% CI)	p	RRR^c^ (99% CI)	p	RRR^c^ (99% CI)	p
Referent category^b^	1.0		1.0		1.0	
History of mental illness only	1.3 (1.1–1.5)	<0.001	1.2 (1.2–1.2)	<0.001	1.3 (1.3–1.4)	<0.001
Un-medicated mental illness	1.4 (0.8–2.5)	0.147	1.0 (0.9–1.2)	0.837	1.0 (0.9–1.2)	0.457
TCAs	1.6 (0.9–2.9)	0.056	1.3 (1.1–1.5)	<0.001	1.7 (1.5–1.9)	<0.001
SSRIs	1.6 (1.1–2.4)	0.001	1.5 (1.3–1.6)	<0.001	2.2 (2.1–2.4)	<0.001
Benzodiazepines	2.0 (1.0–3.8)	0.007	1.6 (1.4–1.9)	<0.001	2.2 (1.9–2.6)	<0.001
Any other single class	3.7 (1.9–7.5)	<0.001	2.0 (1.7–2.5)	<0.001	2.6 (2.1–3.1)	<0.001
Multiple classes	2.0 (1.0–3.7)	0.006	1.6 (1.4–1.9)	<0.001	2.2 (1.9–2.6)	<0.001

a Exposures were depression or anxiety with or without exposures to different classes of antidepressants or anti-anxiety drugs. All categories were mutually exclusive.

b Reference was no history of or current depression or anxiety.

c Relative risk ratio after adjusted for maternal age at the end of pregnancy, household socioeconomic status, maternal smoking status before delivery and body mass index before pregnancy.

TCAs = tricyclic antidepressants; SSRIs = selective serotonin reuptake inhibitors; CI = confidence interval.

Table S1 shows relative risk ratios for all adverse pregnancy outcomes in the whole population of women (512,574 pregnancies in 331,414 women) after adjusting for the number of previous known live births (a proxy of parity). The results were almost identical to the main estimates in Table 3. Table S2 shows the results from the same analysis but in the 146,887 pregnancies that occurred in women registered by age 20 (85,260 women, 26% of the total population). Although power was reduced, relative risk ratios were similar to our main results with almost all risk estimates remaining within the 99% confidence intervals of the estimates in Table 3. Risk estimates for termination did reduce modestly, yet all adverse outcomes still showed increased treatment-associated risks.

### Assessing risks of medication use in women with depression or anxiety only

Compared with pregnancies in women with un-medicated depression or anxiety, women prescribed psychotropic medication had increased risks of all non-live pregnancy outcomes, although most of the results for perinatal death were not statistically significant at the 1% level (Table 4). The greatest effects were again found among women in Group 6 (adjusted RRRs = 2.7, 2.0 and 2.3, 99% CIs 1.1–6.6, 1.6–2.5 and 1.8–2.8 for the risks of perinatal death, miscarriage and termination, respectively).

**Table 4:** Adjusted relative risk ratios of each adverse pregnancy outcome relative to live birth in each antenatal drug exposure category compared with un-medicated antenatal depression or anxiety.

	Perinatal death		Miscarriage		Termination	
Mental illness/drug exposures^a^	n = 111		n = 2,784		n = 3,991	
	RRR^c^ (99% CI)	p	RRR^c^ (99% CI)	p	RRR^c^ (99% CI)	p
Referent category^b^	1.0		1.0		1.0	
TCAs	1.2 (0.5–2.7)	0.651	1.3 (1.1–1.5)	0.001	1.4 (1.2–1.7)	<0.001
SSRIs	1.2 (0.6–2.3)	0.558	1.4 (1.2–1.7)	<0.001	2.0 (1.8–2.3)	<0.001
Benzodiazepines	1.4 (0.6–3.4)	0.305	1.6 (1.3–1.9)	<0.001	1.9 (1.6–2.3)	<0.001
Any other single class	2.7 (1.1–6.6)	0.006	2.0 (1.6–2.5)	<0.001	2.3 (1.8–2.8)	<0.001
Multiple classes	1.4 (0.6–3.3)	0.308	1.6 (1.3–1.9)	<0.001	2.0 (1.6–2.3)	<0.001

a Exposures were depression or anxiety with or without exposures to different classes of antidepressants or anti-anxiety drugs. All categories were mutually exclusive.

b Reference was unmedicated depression or anxiety during the first trimester of pregnancy.

c Relative risk ratio adjusted for maternal age at the end of pregnancy, household socioeconomic status, maternal smoking status before delivery and body mass index before pregnancy.

TCAs = tricyclic antidepressants.

SSRIs = selective serotonin reuptake inhibitors.

CI = confidence interval.

### Assessing effects of continued medication use in pregnancy

Table 5 shows the adjusted RRRs of non-live pregnancy outcomes in pregnant women continuing with each psychotropic medication during the first trimester of pregnancy compared with those who discontinued the medication. There were no increased risks of non-live pregnancy outcomes in women continuing with TCAs during pregnancy compared with those discontinuing them. In contrast, women who continued with SSRIs and benzodiazepines had modest increased risks of miscarriage (RRRs = 1.2 and 1.5, 99% CIs 1.0–1.3 and 1.0–2.1, respectively) as well as termination (RRRs = 1.5 and 1.9, 99% CIs 1.3–1.6 and 1.4–2.6, respectively) compared with those who did not.

**Table 5:** Adjusted relative risk ratios of each adverse pregnancy outcome relative to live birth in pregnancies where women continued psychotropic medication use during the first trimester compared with those where women discontinued use.

Drug exposures^a^	Perinatal death			Miscarriage			Termination		
	n	RRR^b^ (99% CI)	p	n	RRR^b^ (99% CI)	p	n	RRR^b^ (99% CI)	p
**TCAs only (N** c ** = 4,349)**	**22**			**650**			**708**		
Discontinuing (n = 2,708)	12	1.0		396	1.0		434	1.0	
Continuing (n = 1,641)	10	1.5 (0.4–5.2)	0.406	254	1.0 (0.8–1.3)	0.861	274	1.1 (0.9–1.4)	0.387
**SSRIs only (N** c ** = 14,191)**	**69**			**2,069**			**3,090**		
Discontinuing (n = 7,203)	30	1.0		1,005	1.0		1,411	1.0	
Continuing (n = 6,988)	39	1.4 (0.7–2.6)	0.223	1,064	1.2 (1.0–1.3)	0.002	1,679	1.5 (1.3–1.6)	<0.001
**Benzodiazepines only (N** c ** = 3,392)**	**25**			**520**			**654**		
Discontinuing (n = 2,717)	19	1.0		415	1.0		491	1.0	
Continuing (n = 611)	6	1.7 (0.5–6.0)	0.293	105	1.5 (1.0–2.1)	0.004	163	1.9 (1.4–2.6)	<0.001

a Women with exposure to TCAs, SSRIs, or benzodiazepines only during 90 days before conceptions continued or discontinued with the medication during the first trimester of pregnancy.

b Relative risk ratio adjusted for maternal age at the end of pregnancy, household socioeconomic status, maternal smoking status before delivery and body mass index before pregnancy.

c Total exposed pregnancies (ending in live and non-live outcomes).

TCAs = tricyclic antidepressants; SSRIs = selective serotonin reuptake inhibitors; CI = confidence interval.

## Discussion

### Principal findings

The results of our analyses suggest that a history of depression or anxiety is associated with an increased risk of miscarriage and perinatal death, and of elective termination of pregnancy. The use of medication during the first trimester of pregnancy appears to be associated with an increase in these risks, whilst women who discontinue their use of medication at the onset of pregnancy appear to have reduced risks relative to those who do not.

### Strengths and limitations

Our study is the largest and most comprehensive so far to examine the association between maternal depression and anxiety, the use of pharmacological treatments for these illnesses and the risks of perinatal death, miscarriage and termination. Our study is the first to investigate all of these outcomes whilst differentiating between past illness, current illness without medication use, and current medication use stratified by medication class and the number of medication types prescribed. We have also examined the impact of drug discontinuation; to our best knowledge this analysis is novel.

Our large sample size and assessments of significance at the 1% level mean that our findings are unlikely to be due to chance alone. Since perinatal deaths are comparatively rare in the UK population, negative results for these outcomes should be interpreted cautiously as power is somewhat limited and we cannot exclude the possibility that we have failed to detect true risks. However, given the rarity of these events, effects of the observed magnitude would in any case translate to fairly small risks in absolute terms.

Our data were obtained from a UK primary care database and prospectively recorded by primary care physicians, excluding the possibility of recall bias. We may have missed some non-live pregnancy outcomes, such as very early miscarriages and private terminations, however the prevalence of clinically-recognised adverse pregnancy outcomes in our population is similar to UK national estimates. [30]–[32] In addition, we may have missed some women with depression and anxiety who do not report their symptoms to their primary care physicians. Since all pregnant women must be registered with primary care physicians in the UK in order to benefit from antenatal checks and free medication, it is unlikely that a high proportion of women with depression and anxiety (and especially those with prescriptions for psychotropic medication) were not identified. Some women receiving prescriptions may not actually take the medication; this, however, would tend to bias our estimates to the null hypothesis (rather than produce spurious associations). Inevitably in these data, our population of women with depression or anxiety represents those diagnosed and clinically treated and our identification of exposure is therefore pragmatic rather than exhaustive.

We have adjusted for the effects of maternal age, socio-economic deprivation, maternal smoking and maternal BMI. We do not have complete data on these factors, but the absence of any evidence of confounding where data are available suggests that it is unlikely that there is substantial residual confounding where data are missing. We have also adjusted for the number of previous known live births in overall population and in a subset of women registered by age 20 (for whom parity estimates should be accurate). Although there was some evidence of residual confounding as risk estimates did decrease slightly for termination in the subgroup analysis, drug-associated risks for perinatal death and miscarriage remained almost unchanged. It should be noted, however, that this subgroup of women is younger than the overall population, so higher parity numbers and episodes of past depression are less common. Nevertheless, there was no evidence that the patterns of treatment-associated risks particularly with miscarriage and perinatal death were confounded by women's pregnancy history.

We acknowledge that other unmeasured factors might partly explain our results. One particularly important effect that we have not quantified is the severity of disease, whether in terms of symptoms or other measures. It is impossible for us to completely separate the effects of psychotropic drugs from the indications for treatment, and the receipt of medication might imply more severe illness. Pregnant women with more severe mental illness might be more likely to choose a subsequent termination. Since risk estimates were slightly higher for pregnancies ending in terminations than for perinatal death or miscarriage for almost every drug class, it is therefore possible that differing severity of underlying illness does partly explain our findings. However, in the analysis of drug continuation in pregnancy, the differing effect of continuing with SSRIs or benzodiazepines from the effect of continuing with TCAs does suggest some medication-specific (and therefore pharmacological) contribution to the observed increases in risk, although the true effect could be marginal.

### Interpretation in context of previous studies

Our findings of increased risks of miscarriage and perinatal death among women with a history of medicated depression or anxiety during early pregnancy were generally consistent with previous studies. [4]–[8], [10], [11], [20], [22], [23] To some extent, however, our findings also differed from previous work.

A Swedish study [20] found a 70% increased risk, though not statistically significant (adjusted risk ratio = 1.7, 95% CI 0.6–3.6), of stillbirth in women exposed to newer antidepressants (venlafaxine, mirtazapine, miaserin and reboxetine) during the first trimester of pregnancy compared with those without such exposure after adjusting for maternal age, year of birth, parity, maternal smoking and maternal BMI, which is similar to our study. However, there was no increased risk of stillbirth in women exposed to SSRIs (adjusted risk ratio = 0.8, 95% CI 0.5–1.2). Women with a history of depression or anxiety but no medication during pregnancy were included in the referent group for comparison and fewer than 1% of women had received SSRIs (this is under half as many as in our UK population, suggesting differing clinical criteria for issuance of treatment), limiting statistical power, which may partly explain our different findings.

Four prospective cohort studies [8], [33]–[35] investigating women consulting the same teratology information service in Canada found on average a 1.5–2 fold increased risk of miscarriage in women taking TCAs, SSRIs and newer antidepressants such as venlafaxine during the first trimester of pregnancy. All four studies, however, had relatively small sample sizes (the largest being 534) and considerable uncertainty in the estimates. Chambers *et al*. [11] conducted another cohort study in 408 women who contacted a teratology information service in the USA from 1989 to 1995 and did not find statistically significantly increased risks of miscarriages or stillbirths in pregnant women taking fluoxetine during the first trimester compared with those not taking fluoxetine. However, by pooling the results from the six studies, [8], [11], [33]–[36] Hemels and colleagues [36] found a 45% (risk ratio = 1.45, 95% CI 1.19–1.77) increased risk of miscarriage in mothers taking any antidepressants during early pregnancy. Specifically, they found increased risks in women prescribed SSRIs and newer antidepressants, but not TCAs, compared (in contrast with our approach) with women who were not prescribed the respective class of drugs (risk ratios = 1.23, 1.52 and 1.65, 95% CIs 0.84–1.78, 1.17–1.98 and 1.02–2.69 for TCAs, SSRIs, and new antidepressants, respectively). It is important to note, however, that study populations derived from teratology information services likely represent highly selected groups that exclude many exposed women in the general population.

A more recent case-control study including more than half a million pregnant women from Canada [6] found a 68% (95% CI 1.38–2.06) increased risk of miscarriage in women prescribed antidepressants even after adjusting for depression, anxiety, history of medication use during one year before pregnancy and the severity of the illness (defined as the number of days antidepressants prescribed and the number of visits to a psychiatrist in the year before pregnancy). Specifically, they observed a higher risk in women taking SSRIs, but not among those taking TCAs (odds ratios = 1.61 and 1.27, 95% CIs 1.28–2.04 and 0.85–1.91, respectively). These findings suggest a potential pharmaceutical effect with SSRIs but not with TCAs, which is consistent with our own observations. This study also found a three-fold increased risk of miscarriage in women with multiple classes of antidepressants compared with those with one class only (odds ratio = 3.51, 95% CI 2.20–5.61 for at least 2 different classes of antidepressants). Again, the authors did not directly compare un-medicated cases with those prescribed medication, nor did they consider the effects of anxiolytics, such as benzodiazepines, which were also associated with greater risks of non-live pregnancy outcomes in our study.

To our best knowledge, only a few studies have examined the effect of anti-anxiety drugs on non-live pregnancy outcomes. [22], [23], [37], [38] A large American study in the 1970s [38] found a higher, though not statistically significant, risk of perinatal death in women prescribed meprobamate and chlordiazeproxide, and a later case-control study in Sweden [23] showed a 4-fold increased odds of perinatal death in women exposed to benzodiazepines during pregnancy (95% CI 2.0–7.9). A prospective study in Israel [22] examining women who contacted the teratogen information service during pregnancy found higher rates of miscarriage (8.7% vs. 5.2%) in women exposed to benzodiazepines than those exposed to non-teratogenic drugs. None of these studies, however, assessed the impact of women's underlying mental health conditions or other maternal characteristics.

In addition, our finding that women with medicated anxiety or depression during pregnancy are more likely to terminate a pregnancy than those who do not receive medication, is in line with prior research. [9], [11], [22] Unlike miscarriage and perinatal death (which typically occur due to trauma or via some biological mechanism) choosing to have a termination is usually voluntary, occasionally due to in utero identification of a known chromosomal or congenital anomaly or of a potential risk to the foetus or mother if pregnancy continues to term (though such cases are uncommon in the UK population). Compared with the risks of miscarriage and perinatal death, the increases in risks of termination found in our study were much greater, suggesting that women receiving medication for depression or anxiety during pregnancy may be those who suffer the most severe symptoms and consequently feel unable to cope with a child. The discovery of pregnancy when taking psychotropic medications could also contribute to such decisions since women may worry about the adverse impacts on the health of their offspring subsequently. [9] There may also be a marginal degree of reverse causation insofar as the small number of mothers who discover that their foetus exhibits an abnormality may become depressed and commence treatment prior to having a termination. Our findings concur with those of a recent study [9] in Canada including 937 women taking antidepressants during early pregnancy, which found three-fold increased risks of termination in exposed women compared with those unexposed (OR = 3.25, 95% CI 1.48–7.14), but only a 63% increased risk of miscarriage (OR = 1.63, 95% CI 1.24–2.14).

### Summary and implications

Our study has shown increased risks of miscarriage, perinatal death and decisions to terminate a pregnancy in women with anxiety or depression prior to pregnancy and with exposures to psychotropic drugs during the first trimester of pregnancy. We found even greater risks in women with medicated antenatal depression and anxiety compared with those who did not receive medication. Specifically, the risks were greatest among pregnant women prescribed SSRIs, benzodiazepines, and newer but less common drugs, and in those taking multiple drugs. While we cannot rule out some residual confounding by severity of mental illness, our analysis of women who did and did not continue their medication when pregnant implies that longer exposure may be more harmful. Since the risk of developing a new depressive episode during pregnancy in women discontinuing antidepressants remains unclear, [39]–[41] our findings suggest that clinicians and obstetricians should take a cautious approach to drug treatment in pregnant women with mental illness.

## Supporting Information

Table S1 **Sensitivity analyses: Adjusted relative risk ratios of each adverse pregnancy outcome relative to live birth in each antenatal diagnostic and drug exposure category compared with no current/past depression or anxiety, adjusted for number of previous known live births.**
(DOC)

Table S2 **Sensitivity analyses in women with computerised prospective data from age 20 (146,887 pregnancies in 85,260 women; 26% of total population): Adjusted relative risk ratios of each adverse pregnancy outcome relative to live birth in each antenatal diagnostic and drug exposure category, adjusted for number of previous known live births.**
(DOC)
