# Supplementary material for: Live and Non-Live Pregnancy Outcomes among Women with Depression and Anxiety: A Population-Based Study
Source: PLoS One. 2012 Aug 24;7(8):e43462. doi: 10.1371/journal.pone.0043462 (PMC3427383; doi:10.1371/journal.pone.0043462)
Supplement: Table S1 — Sensitivity analyses: Adjusted relative risk ratios of each adverse pregnancy outcome relative to live birth in each antenatal diagnostic and drug exposure category compared with no current/past depression or anxiety, adjusted for number of previous known live births. (DOC) [file pone.0043462.s001.doc]

**Table S1** Adjusted relative risk ratios of each adverse pregnancy outcome relative to live birth in each antenatal diagnostic and drug exposure category compared with no current/past depression or anxiety

|  | **Perinatal death** | | **Miscarriage** | | **Termination** | |
| --- | --- | --- | --- | --- | --- | --- |
| **Mental illness/drug exposures^a^** | n=2,096 | | n=64,510 | | n=75,524 | |
|  | RRR^c^ (99% CI) | p | RRR^c^ (99% CI) | p | RRR^c^ (99% CI) | p |
| Referent category^b^ | 1.0 |  | 1.0 |  | 1.0 |  |
| History of mental illness only | 1.1 (1.0-1.3) | 0.025 | 1.2 (1.2-1.2) | <0.001 | 1.3 (1.3-1.4) | <0.001 |
| Un-medicated mental illness | 1.2 (0.7-2.3) | 0.361 | 1.0 (0.9-1.2) | 0.854 | 1.0 (0.9-1.2) | 0.434 |
| TCAs | 1.6 (0.9-3.1) | 0.051 | 1.3 (1.1-1.5) | <0.001 | 1.7 (1.5-1.9) | <0.001 |
| SSRIs | 1.4 (1.0-2.1) | 0.015 | 1.5 (1.3-1.6) | <0.001 | 2.2 (2.1-2.4) | <0.001 |
| Benzodiazepines | 1.9 (1.0-3.8) | 0.011 | 1.6 (1.4-1.9) | <0.001 | 2.2 (1.9-2.6) | <0.001 |
| Any other single class | 3.5 (1.6-7.3) | <0.001 | 2.0 (1.7-2.5) | <0.001 | 2.6 (2.1-3.1) | <0.001 |
| Multiple classes | 2.0 (1.1-3.8) | 0.004 | 1.6 (1.4-1.9) | <0.001 | 2.2 (1.9-2.6) | <0.001 |

^a^ Exposures were depression or anxiety with or without exposures to different classes of antidepressants or anti-anxiety drugs. All categories were mutually exclusive.

^b^ Reference was no history of or current depression or anxiety

**^c^ Relative risk ratio adjusted for maternal age at the end of pregnancy, number of previous known live births, household socioeconomic status, maternal smoking status before delivery and body mass index before pregnancy**

TCAs=tricyclic antidepressants; SSRIs=selective serotonin reuptake inhibitors; CI=confidence interval
